# The effects of crocin on psychological parameters in patients under methadone maintenance treatment: a randomized clinical trial

**DOI:** 10.1186/s13011-019-0198-1

**Published:** 2019-02-22

**Authors:** Anahita Khalatbari-mohseni, Hamid Reza Banafshe, Naghmeh Mirhosseini, Zatollah Asemi, Amir Ghaderi, Abdollah Omidi

**Affiliations:** 1 Department of Addiction studies, School of Medical, Kashan University of Medical Sciences, Kashan, Iran; 2 Department of Pharmacology, School of Medicine, Kashan University of Medical Sciences, Kashan, Iran; 3 Physiology Research Center, Kashan University of Medical Sciences, Kashan, Iran; 4 School of Public Health, University of Saskatchewan, Saskatoon, SK Canada; 5 Research Center for Biochemistry and Nutrition in Metabolic Diseases, Kashan University of Medical Sciences, Kashan, I.R. Iran; 6 Department of clinical psychology, School of Medicine, Kashan University of Medical Science, Kashan, Iran

**Keywords:** Crocin, Psychological parameters, Methadone maintenance treatment

## Abstract

**Background:**

Methadone maintenance treatment (MMT) might be associated with the symptoms of depression and anxiety, sleep disturbances and sexual dysfunctions. This study was designed to determine the effects of crocin on psychological parameters in patients under MMT.

**Methods:**

Patients under MMT were randomly allocated into two groups to receive either 30 mg/day crocin (2 plus crocin tablet, 15 mg BID) (*n* = 25) or placebo (2 tablets per day, 15 mg BID) (*n* = 25), one hour after taking food, for 8 weeks. Psychological parameters were evaluated at baseline and end of the trial to determine related associations between crocin and patients’ mental health status.

**Results:**

After 8-week intervention, crocin significantly decreased Beck Depression Inventory (b − 6.66; 95% CI, − 9.88, − 3.45; *P* < 0.0001), Beck Anxiety Inventory (b − 4.35; 95% CI, − 5.94, − 2.75; P < 0.0001), general health questionnaire (b − 4.45; 95% CI, − 7.68, − 1.22; *P* = 0.008) and Pittsburgh Sleep Quality Index (b − 2.73; 95% CI, − 3.74, − 1.73; P < 0.0001) in patients under MMT, compared with the placebo. Crocin also significantly improved International Index of Erectile Functions (b 4.98; 95% CI, 2.08, 7.88; *P* = 0.001) rather than placebo.

**Conclusion:**

Our findings indicated that taking crocin for 8 weeks by patients under MMT had beneficial effects on their mental health status. Crocin can be recommended as an adjunct to methadone in opioid withdrawal protocols because of the ability to improve the quality of life and decrease opioids side effects in these patients.

This trial was registered in the Iranian website for clinical trials registry as http://www.irct.ir: IRCT2017110537243N1.

**Clinical trial registration number:**

www.irct.ir: http://www.irct.ir: IRCT2017110537243N1.

## Background

Methadone is well-known for treating opioids dependence and managing chronic pain among patients suffering from withdrawal syndrome [1]. Being the most cost-effective treatment for opioid dependence, interest in adding it to opioid withdrawal protocols [2, 3] is growing. Methadone maintenance treatment (MMT) might improve social functioning and quality of life among patients with chronic dependence [4]. However, despite the practical implementation of MMT, many challenges and hurdles remained unsolved. There are evidence demonstrating the association between substance dependence and comorbid conditions, specifically psychological syndromes [5]. However, MMT also might be associated with depression and anxiety [6], sleep disturbances [7] and sexual dysfunctions [8] among patients suffering from withdrawal syndrome.

*Crocus sativus L*., commonly known as saffron, belongs to iridaceous family [9]. Several crops are available in France, New Zealand, Switzerland, the United States, England, and other countries some of them organically grown. In the United States, Pennsylvania Dutch saffron known for its “earthy” notes is marketed in small quantities [10]. Saffron contains several compounds such as safranal, picrocrocin and crocin, the latter is the main antioxidant in saffron working as a dye [11]. Saffron and mainly crocin are effective antidepressant and anti-anxiety agents [12], as well as sexual motivator [13], memory enhancer and sedative which are used for the treatment of different central nervous system disorders [14]. The beneficial effects of crocin on psychological parameters have been shown in patients not taking MMT. Talaei et al. [12] have documented that consuming 30 mg/day crocin for 4 weeks significantly improved mental health parameters including depression, anxiety, general health, and mood disorders in patients suffering from major depressive disorder (MDD). Current evidence demonstrating that saffron administration, at a dosage of 28 mg/day for 4 weeks, had beneficial effects on mood, anxiety, and stress management in individuals self-reporting low mood, yet sleep quality did not improve [15]. Recent studies have shown that daily oral consumption of saffron capsules improves sleep quality in diabetic patients [16], and sexual dysfunction among women [13].

Saffron and crocin may improve the function of central nervous system and mental health status through regulating the synthesis of chemical neurotransmitters in the brain, including dopamine, norepinephrine, and serotonin (5- hydroxyltryptamine) [17–19]. To our best knowledge, evidence demonstrating the effects of crocin on improving psychological symptoms in patients under MMT are scarce. So, in the present placebo-controlled clinical trial, we aimed to determine the effects of crocin on psychological symptoms in patients undergoing MMT.

## Methods

### Preparation of crocin tablets

Saffron stigmas were purchased from Novin Saffron Co. (Mashhad, Iran). Crocin was extracted and crystallized from saffron stigmas, using a previous published protocol [20]. Crocin and placebo were similarly formulated into film-coated tablets by the Department of Pharmaceutics, School of Pharmacy in Mashhad University of Medical Science. Each tablet contained 15 mg crocin or placebo.

### Study design and participants

This randomized, double-blinded, placebo-controlled trial (RCT) was registered in the Iranian website for clinical trials registry (http://www.irct.ir: IRCT2017110537243N1 (one of the Primary Registries in WHO Registry Network, with the collaboration of both Ministry of Health and Medical Education. This trial was conducted following Declaration of Helsinki; informed consent was signed by all participants prior to intervention. The subjects were recruited in the Referral center (the Golabchi Clinic) for patients under MMT subjects in Kashan-Iran within a month starting from November 1, 2017. Then, 50 patients, aged 18–60 years, taking MMT were intervened for two months, starting from December 5, 2017. All patients were examined by a psychiatrist according to the fourth revision of the Diagnostic and Statistical Manual of Mental Disorders (DSM-IV). Individuals with the following criteria were included in the trial: confirmed diagnosis of substance dependency based on DSM-IV, the age range of 18–60 years, patients’ willingness to participate in the intervention. The main exclusion criteria were as followed: taking crocin, multivitamin-mineral and antioxidant supplements during the last 3 months before the intervention initiation, history of metabolic diseases including diabetes, hypertension, thyroid and cardiovascular disease.

### Study protocol

Patients under MMT were randomly allocated into two groups to receive either 30 mg/day crocin (2 plus crocin tablet, 15 mg BID) (*n* = 25) or placebo (2 tablets per day, 15 mg BID) (*n* = 25), one hour after taking food, for 8 weeks. Due to lack of evidence about the appropriate dosage of crocin usage in cases under MMT, we used the above-mentioned dose of crocin based on a previous published study in subjects with major depressive disorder (MDD) [12]. A trained staff randomized the subjects using computer-generated random numbers, at the clinic. Randomization and allocation were concealed to the researchers and participants until the completion of final analyses. The crocin and the placebo tablets were prepared in the same shape, color, size, texture and odor, and each tablet container had a random code number for this double-blinded trial. The tablets were ordered to College of Pharmacy, Mashhad University of Medical Sciences. Thus, participants, physician and other investigators were all blind to the treatment group assignment.

Study participants were instructed to have their regular physical activity and not taking any additional supplements during the 8-week intervention. The compliance rate was monitored by sending a brief daily reminder to the participants’ cellphone to take the supplement and counting the left tablets in returned containers. All participants completed a 3-day food record and a physical activity form prior to intervention, at weeks 2, 4 and 6 and end of the trial. Daily macro- and micro-nutrient intakes were calculated by analyzing food data using nutritionist IV software (First Databank, San Bruno, CA). In this trial, physical activity was indicated as metabolic equivalents (METs) in hours per day [21].

### Safety

Participants were asked about any adverse events or complaint during the trial. The symptoms were checked and recorded at baseline and each follow-up visit. Probable side effects were checked and recorded via every day follow-up call and the psychiatrists and physician in charge were responsible for continuing or discontinuing the intervention.

### Measurements

#### Anthropometric measures

Participants’ weight and height were measured, after an overnight fasting, using a standard calibrated scale (Seca, Hamburg, Germany) at baseline and end of the intervention. Body Mass Index (BMI) was calculated as weight in kg divided by height in meters squared.

#### Clinical measures

Psychological outcomes were assessed using Beck Depression Inventory − 21 (BDI-21), General Health Questionnaire − 28 (GHQ-28), Beck Anxiety Inventory − 21 (BAI-21), The Pittsburgh Sleep Quality Index (PSQI) and Internal Index for Erectile Function (IIEF-15). Beck Depression Inventory is the reliable and valid scale of self-rating depression which is commonly used in clinical settings. It consists of 21 items including emotional, behavioral, and somatic symptoms. Beck Depression Inventory scores between 0 and 9, 10–19, and 20–29 stands for normal, mild, and moderate depression respectively. While, the score of 30 and over is indicative of major depression [22]. General Health Questionnaire is a screening device, suitable for all ages except children, which is used to detect minor psychiatric disorders. It consists of 28 items for the diagnosis of somatic symptoms, anxiety and insomnia, social dysfunction, and severe depression; total score ranges from 28 to 112 [23] with higher scores representing the poorer psychological condition of patients. Beck Anxiety Inventory is a brief measure of anxiety with a focus on its somatic symptoms. It consists of 21 items, describing emotional, physiological, and cognitive symptoms of anxiety, which measures the severity of anxiety in adults and adolescents. The scores of 0–21, 22–35, and 35–63 indicate mild, moderate, and severe anxiety respectively [24]. The Internal Index for Erectile Function (IIEF) evaluates men’s sexual function. It consists of 15 questions in five dimensions of sexual desire, orgasm, erection, sexual satisfaction, and overall satisfaction. The total score ranges between 0 and 75; the higher the score the better the sexual functioning. Scores of 0–10, 11–16, 17–21, and 22–25 are indicative of severe, average, average to mild, and mild sexual dysfunction; scores of 26–30 indicate normal sexual function [25]. The Pittsburgh Sleep Quality Index (PSQI) is an applicable instrument used to measure the quality and pattern of sleep in adults. It differentiates “poor” from “good” sleep quality by measuring seven components of sleep including: subjective sleep quality, sleep latency, sleep duration, habitual sleep efficiency, sleep disturbances, use of sleeping medications, and daytime dysfunction over the last month [26].

### Sample size

We used RCTs sample size formula, with type one (α) error to be 0.05 and the power 80%, to calculate sample size. Based on a previous study [12], a standard deviation (SD) of 6.1 and a difference in mean (d) of 11.45 were used considering BDI as the key variable. Based on a previous study, using formula for clinical trial sample size calculation, 25 individuals should have been enrolled in each group. Assuming a dropout of 20% individuals per group, the final sample size was considered to be 30 participants in each intervention group.

### Statistical analysis

The Kolmogorov-Smirnov test was done to determine the normality of data. To detect the differences in anthropometric measures, nutrient intakes and psychological parameters between treatment groups, we used independent-samples *t*-test. Multiple linear regression models were applied to determine treatment effects on study outcomes after adjusting for confounding parameters including; age, and BMI. The effect sizes were presented as the mean differences with 95% confidence intervals. Differences in proportions were evaluated by Chi square test. *P*-values < 0.05 were considered statistically significant. All statistical analyses were done using the Statistical Package for Social Science version 18 (SPSS Inc., Chicago, Illinois, USA).

## Results

**Fig. 1:**
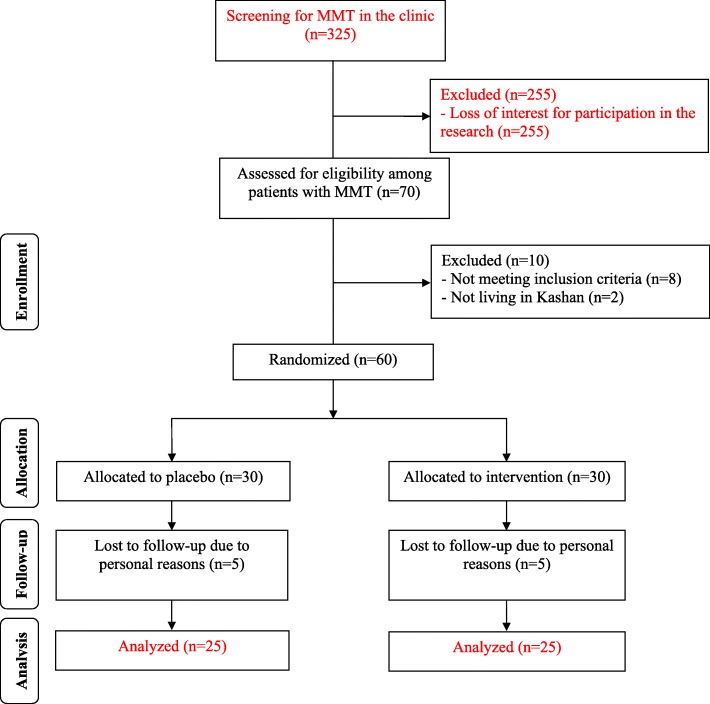
Summary of patient flow

Five patients from each intervention group withdraw from the trial due to personal reasons. Finally, 50 participants [crocin (*n* = 25) and placebo (*n* = 25)] completed the study **(**Fig. 1**).** The compliance rate in this study was high; more than 90% of capsules were taken throughout the intervention in both groups. No serious side effects were reported following the consumption of crocin. However, grade 1 side effects were reported in 6 patients using crocin [headache (*n* = 2), insomnia (*n* = 1), nausea (*n* = 2) and dyspnea (*n* = 1)] and 2 patients in the placebo group [headache (*n* = 1) and nausea (*n* = 1)], which did not lead to excluding any patients from the study.

Education level, marital status, job, other medication consumption, methadone dose, the duration of MMT and mean age, height, weight and BMI were not significantly different between crocin and placebo groups **(**Table 1**).**

**Table 1:** General characteristics of the study participants^1^

	Placebo group (*n* = 25)	Crocin group (*n* = 25)	*P* ^2^
Age (y)	41.4 ± 8.8	40.1 ± 9.3	0.63
Height (cm)	171.2 ± 7.5	171.6 ± 8.4	0.84
Weight at study baseline (kg)	70.1 ± 8.5	68.9 ± 12.1	0.68
Weight at the end-of-trial (kg)	69.7 ± 8.6	69.0 ± 11.7	0.81
Weight change (kg)	−0.4 ± 1.6	0.08 ± 1.4	0.23
BMI at study baseline (kg/m^2^)	23.9 ± 2.9	23.3 ± 3.6	0.51
BMI at the end-of-trial (kg/m^2^)	23.8 ± 3.0	23.4 ± 3.6	0.66
BMI change (kg/m^2^)	−0.1 ± 0.5	0.05 ± 0.5	0.19
Education (%)			
Illiterate	5 (20)	7 (28)	
Elementary	5 (20)	7 (28)	
Intermediate	12 (48)	10 (40)	0.58†
Diploma	2 (8)	0 (0)	
High educated	1 (4)	1 (4)	
Marital status (%)			
Single	8 (32)	5 (20)	
Married	5 (20)	8 (32)	0.50†
Widow/Divorced	12 (48)	12 (48)	
Job (%)			
Unemployed	16 (64)	18 (72)	
Employed	2 (8)	1 (4)	0.76†
Others	7 (28)	6 (24)	
Use of other drugs (%)			
None	20 (80)	19 (76)	
Benzodiazepine	2 (8)	3 (12)	0.89^†^
Antidepressants (SSRIs)	3 (12)	3 (12)	
Methadone dose (mL/d)	19.2 ± 6.2	17.2 ± 5.9	0.25
Duration of MMT (y)	6.2 ± 2.6	5.5 ± 1.9	0.33

^a^Data are mean ± SDs

^b^Obtained from independent *t*-test

^c^Obtained from Pearson Chi-square test

*SSRIs* selective serotonin reuptake inhibitors

Macro- and micro-nutrient intakes, calculated based on 3-days food records, were not significantly different between intervention groups (Data not shown).

**Fig. 2:**
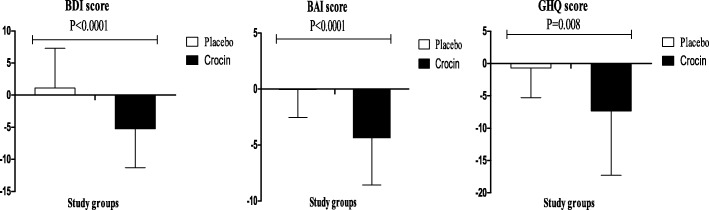
Change (means± SDs) in BDI score, BAI score and GHQ score in patients under MMT who were candidate for receiving crocin supplements and placebo. *P* value was obtained from independent *t*-test. *N* = 25 in each group

**Fig. 3:**
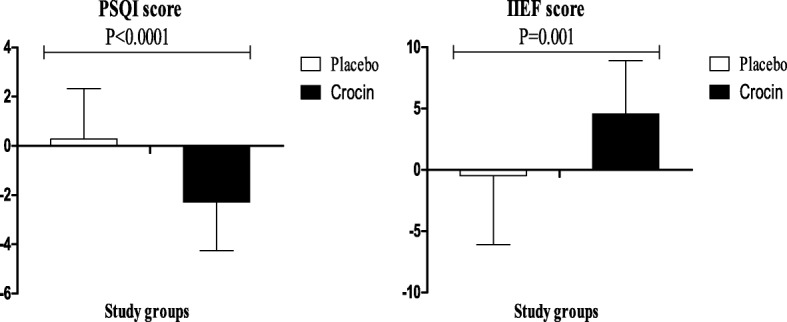
Change (means± SDs) in PSQI score and IIEF score in patients under MMT who were candidate for receiving crocin supplements and placebo. P value was obtained from independent *t*-test. N = 25 in each group. BDI, Beck Depression Inventory; BAI, Beck Anxiety Inventory; GHQ, general health questionnaire; IIEF, International Index of Erectile Functions; MMT, methadone maintenance treatment; PSQI, Pittsburgh Sleep Quality Index

Crocin significantly decreased BDI (b − 6.66; 95% CI, − 9.88, − 3.45; *P* < 0.0001), BAI (b − 4.35; 95% CI, − 5.94, − 2.75; *P* < 0.0001), GHQ (b − 4.45; 95% CI, − 7.68, − 1.22; *P* = 0.008) and PSQI (b − 2.73; 95% CI, − 3.74, − 1.73; *P* < 0.0001) in patients undergoing MMT, compared with the placebo. In addition, crocin significantly improved IIEF in these patients (b 4.98; 95% CI, 2.08, 7.88; *P* = 0.001) compared with the placebo **(**Table 2**).** Changes in BDI score, BAI score, GHQ score, PSQI score and IIEF score in patients under MMT receiving crocin supplements and placebo are presented in Figs. 2 & 3**.**

**Table 2:** The effect of crocin on psychological parameters in methadone maintenance treatment patients

Variables	Placebo group (*n* = 25)		Crocin group (*n* = 25)		Difference in outcome measures between crocin and placebo treatment groups^a^	
	Baseline	Week 8	Baseline	Week 8	β (95% CI)	*P* ^b^
BDI score	23.6 ± 6.6	24.7 ± 7.3	22.8 ± 5.4	17.6 ± 5.0	−6.66 (−9.88,-3.45)	< 0.0001
BAI score	19.2 ± 4.0	19.2 ± 3.5	19.7 ± 6.6	15.3 ± 5.2	−4.35 (−5.94, −2.75)	< 0.0001
GHQ score	35.6 ± 13.0	34.4 ± 12.23	34.8 ± 11.5	29.3 ± 10.2	−4.45 (−7.68, −1.22)	0.008
PSQI	6.7 ± 2.3	7.0 ± 2.6	6.4 ± 2.3	4.1 ± 1.8	−2.73 (−3.74, −1.73)	< 0.0001
IIEF	22.0 ± 11.8	21.6 ± 12.1	21.0 ± 16.7	25.64 ± 16.5	4.98 (2.08, 7.88)	0.001

Data are mean ± SDs

^a^“Outcome measures” refers to the change in values of measures of interest between baseline and week 8. β [difference in the mean outcomes measures between treatment groups (crocin group = 1 and placebo group = 0)]

^b^Obtained from multiple regression model (adjusted for baseline age and BMI)

*BDI* Beck Depression Inventory, *BAI* Beck Anxiety Inventory, *GHQ* general health questionnaire, *IIEF* International Index of Erectile Functions, *PSQI* Pittsburgh Sleep Quality Index

## Discussion

We evaluated the impacts of 8 weeks crocin intake on psychological parameters in patients under MMT. Our findings documents that crocin in patients under MMT significantly improves their depression, anxiety, general health, sleep quality and erectile functions. Based on these findings, crocin may be recommended as an adjunct therapy for opioid-dependent patients under treatment with MMT. To our best knowledge, this study for the first evaluated the effects of crocin on psychological symptoms of patients undergoing MMT.

### Effects on depression, anxiety and general health

Methadone maintenance treatment is usually associated with some complications including depression, anxiety and poor general health [5, 6, 27–29]. We found that taking crocin by the patients who were under MMT for 8 weeks improved depression, anxiety and general health. Nowadays herbal medicine has attracted lots of interest in the treatment of psychiatric problems such as depression, anxiety and mental disorder. There are studies confirming the beneficial impacts of crocin administration on depression, anxiety and general health in individuals not undergoing MMT. In the study conducted by Talaei et al. [12], crocin intake at a dosage of 30 mg/day for 4 weeks improved depression, anxiety, general health and mood disorder in patients had been diagnosed with MDD. In another study, administration of saffron extract for 6 weeks to depressed patients helped with the treatment of mild to moderate depression [30]. Moreover, following saffron administration at a dosage of 28 mg/day for 4 weeks, the median value of anxiety and stress were significantly decreased in healthy adults [15]. In addition, taking saffron at a dosage of 15 mg/Bid for 8 weeks by mothers suffering from mild-to-moderate postpartum depressive disorder significantly reduced depression and anxiety [31]. Also, in an animal study conducted by Hosseinzadeh et al. [18], the petal and stigma of saffron were introduced as antidepressant in rat and mice models. All of these effects may be attributed to a synergistic action of different constituents in saffron such as crocin, picrocrocin, safranal and flavonoids [12]. The accurate mechanism of action for crocin in the brain and its impacts on depression and anxiety parameters is not completely understood. Adjunctive therapy using crocin may provide powerful antioxidant effects and prevent free radical induced damage in the brain and subsequently improve mental health status [32].

### Effects on sleep quality

Sleep disorders are common among patients undergoing MMT and the underlying reason may be due to drug abuse related signs and other psychiatric co-morbidities [33, 34]. Our study demonstrated that crocin administration to patients under MMT for 8 weeks significantly had beneficial effects on their sleep quality. Little is known about the effects of crocin on sleep quality in patients under MMT. In a study conducted by Kell et al. [15], saffron at dosages of 22 mg/day and 28 mg/day for 4 weeks significantly improved the quality of sleep in healthy adults. In another study, after saffron intake at a dosage of 300 mg/day for 7 days there were beneficial effects in reducing anxiety and improving the quality of sleep among diabetic patients [35]. Also, in an animal study by Masaki et al. [36], crocin increased the total time of non-REM sleep in mice. Unlike, there are animal studies showing that crocin administered intraperitoneal, did not affect hypnotic activity and anxiety [37]. The effects of saffron on sleep quality are likely associated with the crocin and safranol compounds of saffron [38]. Crocin and safranal likely affect dopaminergic and serotonergic system and nor-epinephrine reuptake inhibition [39]. Also, crocin may modulate the histaminergic or cholinergic arousal system [36]. Saffron has similar activity to hypnotic drugs. Similar to diazepam as a benzodiazepine, it has anxiolytic, analgesic, and sleeping effects [39].

### Effects on sexual functions

Our findings showed that compared with the placebo; taking crocin for 8 weeks by patients under MMT had improved sexual functions. Previous studies have demonstrated higher prevalence of sexual dysfunction among patients undergoing Methadone Maintenance Treatment (MMT) [8]. Several studies have evaluated the effects of crocin intake on sexual functions in individuals not undergoing MMT, though data on the effects of crocin administration on sexual function in patients under MMT are scarce. Saffron administration significantly improves erectile dysfunction in diabetic patients [40]. In a study conducted by Shamsa et al. [20], oral saffron administration at a dose of 200 mg/day for 10 days improved erectile function in males suffering from erectile dysfunction [41]. In addition, taking saffron (15 mg twice/day) for four weeks by married men with major depressive disorder (MDD) suffering from fluoxetine-related sexual dysfunction had promising effects on sexual dysfunction with acceptable patient’s tolerance [42]. Also, in an animal study by Hosseinzadeh et al. [43], administered safranal, crocin, and sildenafil to normal male rats increased sexual desire and frequency of erection in groups receiving Sildenafil and crocin. However, our findings were inconsistent with the results from previous studies on treating erectile dysfunction in men naïve to treatment [44]. This inconsistency may be attributed to the quality of saffron and its cultivation site, the dosage used, and the formula used for the prepared medication. Saffron appears to interact with several neurotransmitter systems [45–47]. Moreover, nitric oxide and opioid system play important roles in sexual function, and saffron seems to interact with both of them [48–50]. Whether the underlying mechanisms of these effects lead to the beneficial sexual effects of saffron certainly requires additional studies [17, 51, 52].

#### Limitations

The present study had some limitations. The duration of intervention was short. Long-term interventions might lead to better effects on psychological parameters. In addition, we did not evaluate the effects of crocin on biomarkers of inflammation, oxidative stress, and its related gene expression. Also, we could not investigate the pain in patients under MMT. Therefore, investigation of pain caused by quitting is suggested in future studies.

## Conclusions

Overall, taking crocin by patients under MMT had beneficial effects on depression, anxiety, general health, sleep quality and sexual functions. Crocin can be recommended as an adjunct to methadone in opioid withdrawal protocols which improves quality of life and diminishes opioids side effects.

## Abbreviations

BAI: Beck Anxiety Inventory
BDI: Beck Depression Inventory
GHQ: General health questionnaire
IIEF: International Index of Erectile Functions
PSQI: Pittsburgh Sleep Quality Index
